# Focal epilepsy with sensory seizures associated with arginine:glycine amidinotransferase deficiency: A clinical and advanced magnetic resonance imaging study

**DOI:** 10.1111/epi.18442

**Published:** 2025-05-05

**Authors:** Francesco Fortunato, Roberta De Fiores, Ilaria Sammarra, Maria Celeste Bonacci, Maria Eugenia Caligiuri, Miriam Sturniolo, Iolanda Martino, Antonio Gambardella

**Affiliations:** ^1^ Department of Medical and Surgical Sciences, Institute of Neurology Magna Græcia University Catanzaro Italy; ^2^ Department of Medical and Surgical Sciences, Neuroscience Research Center Magna Græcia University Catanzaro Italy

**Keywords:** arginine:glycine amidinotransferase, climate change, epilepsy, seizure

## Abstract

We aim to determine whether epilepsy can be considered part of the arginine:glycine amidinotransferase (AGAT) deficiency syndrome phenotype and to identify its associated electroclinical signatures. We reviewed clinical data from our center, identifying individuals with AGAT deficiency. Each individual underwent a dedicated epilepsy assessment with electroencephalography and 3‐T brain magnetic resonance imaging (MRI). Additionally, 30 age‐ and sex‐matched healthy controls (18 females, 28.2 ± 3.7 years old) were recruited for advanced MRI analysis. A family with four affected members carrying homozygous AGAT c.446>A:p.Trp149* variant was identified. Two individuals had focal epilepsy with sensory seizures characterized by a prominent “tingling” sensation. Three experienced febrile seizures plus and marked temperature sensitivity. Corpus callosum dysmorphisms were observed in three cases. Despite creatine supplementation, cortical thickness was significantly reduced across multiple brain regions compared to controls, as indicated by *Z*‐scores. A brain map of AGAT mRNA expression revealed lower expression in the parieto‐occipital areas. Our findings suggest that focal epilepsy with sensory seizures and temperature‐related seizures may be part of the AGAT deficiency spectrum. Furthermore, significant brain atrophy was demonstrated, despite creatine supplementation. The sensory‐predominant epilepsy phenotype aligns with observed atrophy and AGAT‐mRNA regional expression patterns, supporting its biological plausibility.

## 1 INTRODUCTION

Creatine deficiency syndromes are a group of rare monogenic neurodevelopmental disorders caused by impaired creatine synthesis or transport, involving two enzymes, arginine:glycine amidinotransferase (AGAT) and guanidinoacetate methyltransferase (GAMT), as well as the creatine transporter (SLC6A8).^1^, ^2^, ^3^, ^4^

Epilepsy has been reported as the second most common phenotype in GAMT deficiency and is also a frequent manifestation of SLC6A8 deficiency.^4^ To date, no cases with epilepsy have been reported in association with AGAT deficiency. Febrile seizures have been reported in one proband of the index family,^1^, ^4^, ^5^, ^6^ and an individual with a homozygous c.553G>C(p.A185P) variant manifested a single isolated seizure.^7^

AGAT has not been included in the well‐established curated Gene4Epilepsy_v2024_09 panel,^8^ whereas GAMT and SLC6A8 have. Furthermore, AGAT has been classified as a "red gene" in the Early Onset or Syndromic Epilepsy (v7.46) panel from Genomics England (GEL),^9^ signifying a lack of evidence supporting its role as a gene for epilepsy.

A formal diagnosis of creatine deficiency offers a unique opportunity for precision medicine, as early creatine supplementation can improve neurodevelopmental trajectories.^3^, ^6^ Daily oral creatine supplementation may restore up to 80%–90% of brain creatine levels in AGAT deficiency but does not achieve full restoration as documented by brain magnetic resonance spectroscopy (MRS).^1^, ^3^

With these premises in mind, we ask: (1) is epilepsy part of AGAT deficiency syndrome? (2) does epilepsy in AGAT deficiency have specific electroclinical signatures? and (3) is there evidence of neurodegeneration in adults with AGAT deficiency despite creatine supplementation?

## 2 MATERIALS AND METHODS

### 2.1 Ethics statement

This research was approved by the relevant ethics committee. Written informed consent for research use of clinical–genetic data was obtained from individuals or legal guardians for those with intellectual disability.

### 2.2 Cohort selection and clinical assessment

From January 2024 to January 2025, we reviewed clinical and genetic data from our outpatient epilepsy clinic at Magna Græcia University of Catanzaro, Italy, identifying individuals or families with AGAT deficiency, characterized by biallelic AGAT pathogenic variants.

The main inclusion criterion was a confirmed genetic diagnosis of AGAT deficiency. Exclusion criteria were as follows: suspected AGAT deficiency based on brain MRS without genetic confirmation; creatine deficiency due to other relevant causes such as GAMT or SLC6A8 pathogenic variants; and neurodevelopmental disorders with AGAT variants that did not fulfill the American College of Medical Genetics and Genomics criteria for "pathogenic" or "likely pathogenic" variants.^10^

All subjects underwent a dedicated epilepsy assessment, including electroencephalography (EEG) and 3‐T brain magnetic resonance imaging (MRI) protocol with FreeSurfer. Detailed protocol is reported in Data S1.

### 2.3 Control cohort

Thirty healthy individuals (18 females, 28.2 ± 3.7 years old) as a separate age‐ and gender‐matched control cohort were recruited for advanced MRI analysis. This cohort was previously shared in the ENIGMA‐Epilepsy consortium^11^ and a recent publication.^12^

### 2.4 Allen Human Brain Atlas: Brain expression map of the AGAT gene

We used the Allen Human Brain Atlas (https://human.brain‐map.org) as the primary source of AGAT expression data to obtain a brain map of expression of the AGAT gene via a validated pipeline.^13^ Further details are reported in Data S2.

### 2.5 Statistical analysis

Statistical analysis was performed with R software (v4.4.2). Normality was assessed via the Shapiro–Wilk test and homoscedasticity with Levene test. Variables were normally distributed for *p* > .05. For AGAT cases, *Z*‐scores were calculated for each region of interest (ROI) in the quantitative MRI analysis. Brain *Z*‐score maps were generated using residuals from the control cohort via the following general linear model: ROI ~ age + gender + intracranial volume.

## 3 RESULTS

### 3.1 Cohort description

We identified a family originating from Calabria (Italy) with four related members affected by a neurodevelopmental disorder associated with a homozygous pathogenic AGAT variant c.446>A:p.Trp149*. This family has already been described in literature.^1^, ^3^, ^5^, ^6^ The updated pedigree of the family and their comprehensive clinical data are reported in Figure S1 and Table S1.

**FIGURE 1:**
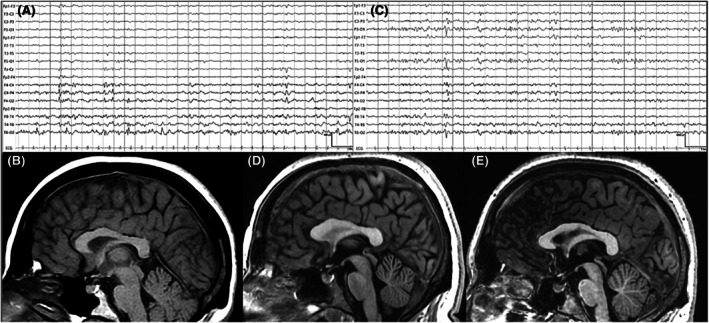
Electroencephalographic (EEG) and qualitative brain 3‐T magnetic resonance imaging (MRI) features. Interictal bipolar anterior–posterior EEG of the proband (A) and the proband's brother (C) showing prominent temporoparieto‐occipital interictal epileptiform discharges. Three‐dimensional T1‐weighted midsagittal brain MRI of the proband (B), the proband's brother (D), and the proband's sister (E) showing subtle dysmorphic features of the corpus callosum.

The proband (IV‐2) is a 30‐year‐old woman with moderate intellectual disability. She experienced simple febrile seizures from 12 months to 12 years old. At 30 years, she was referred to our epilepsy clinic for focal seizures, starting with a brief biparietal headache, followed by left arm tingling spreading to the left hemilabial region. Seizures could progress to left hemiface clonic movements, impaired awareness, and postictal confusion. She reported two motor seizures and several "sensory" seizures. Neurological and formal neuropsychological examination revealed microcephaly, global memory impairment, and language delay. EEG showed right temporoparietal–occipital spike and wave discharges (Figure 1A). Brain 3‐T MRI was unremarkable except for dysmorphic corpus callosum (Figure 1B). She had been on 3 g/day creatine since the age of 4 years. Lamotrigine (100 mg/day) was introduced, achieving seizure freedom after 6 months.

The proband's 20‐year‐old brother (IV‐3) began creatine therapy at four months. He did not exhibit intellectual disability. He had three febrile tonic–clonic seizures at the ages of 6, 10, and 12 years, respectively. At 12 years old, he experienced focal aware sensory seizures like the proband, sometimes ending in prolonged dysphasia or, rarely, progressing to focal‐to‐bilateral tonic–clonic seizures. He occasionally reported tongue tingling at seizure onset. Neurological examination was unremarkable. EEG revealed parieto‐occipital sharp‐wave discharges (Figure 1C). Brain 3‐T MRI showed mild supratentorial leukoencephalopathy with a dysmorphic corpus callosum (Figure 1D). IV‐3 was treated with carbamazepine 800 mg/day resulting in seizure freedom.

The proband's 33‐year‐old sister (IV‐1) had moderate intellectual disability and had been on creatine since the age of 5 years. She never experienced febrile or afebrile seizures. Brain 3‐T MRI showed a dysmorphic corpus callosum (Figure 1E).

The proband's 24‐year‐old paternal nephew (IV‐4) had mild intellectual disability and started creatine supplementation at the age of 2 years. He had multiple febrile seizures throughout childhood with an unclear age at onset. He did not receive brain 3‐T MRI.

### 3.2 Brain 3‐T MRI advanced analysis

**FIGURE 2:**
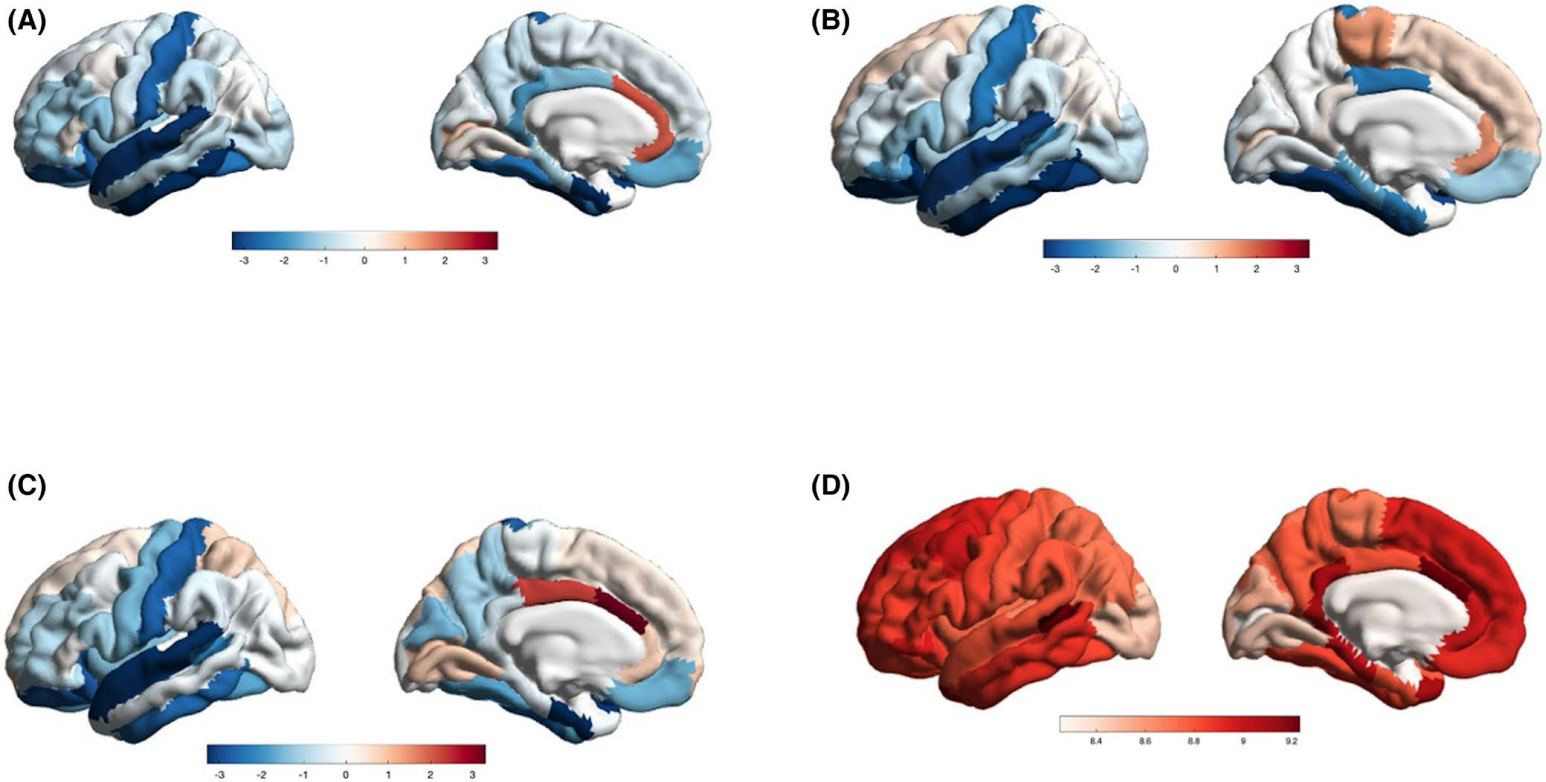
Three‐dimensional (3D) brain magnetic resonance imaging *Z*‐score maps for the three cases with arginine:glycine amidinotransferase (AGAT) deficiency (A–C) and a 3D brain map showing mRNA AGAT expression levels. The figures were created using MATLAB, and the Desikan–Killiany Atlas was used for all images to generate cortical regions of interest, which were averaged between the left and the right hemispheres. (A–C) Color scales indicate *Z*‐scores of cortical thickness lower than controls (blue scale) or higher (red scale). (D) Color scale indicates AGAT mRNA expression levels from the Allen Human Brain Atlas. A more intense red represents higher expression.

We generated three individual brain *Z*‐score maps for the three cases with brain 3‐T MRI, comparing them to 30 matched controls (Figure 2). All cases showed reduced mean cortical thickness (IV‐2: *Z*‐score = −1.60, IV‐1: *Z*‐score = −1.26, IV‐3: *Z*‐score = −1.17) and, in epilepsy cases, marked cortical surface reduction (IV‐2: *Z*‐score = −3.55, IV‐3: *Z*‐score = −3.78). The postcentral parietal cortex was notably thinner (IV‐2: *Z*‐score = −2.69, IV‐1: *Z*‐score = −2.65, IV‐3: *Z*‐score = −2.40). Similar reductions were observed in the entorhinal cortex (IV‐2: *Z*‐score = −2.69, IV‐1: *Z*‐score = −2.65, IV‐3: *Z*‐score = −2.40), superior temporal cortex (proband: *Z*‐score = −3.23, proband's sister: *Z*‐score = −3.18, proband's brother: *Z*‐score = −3.08), inferior temporal cortex (proband: *Z*‐score = −3.54, proband's sister: *Z*‐score = −3.22, proband's brother: *Z*‐score = −2.23). The generated three‐dimensional brain map of AGAT expression levels showed an anterior–posterior gradient, indicating a lower expression in the parieto‐occipital regions (Figure 2D). For additional details, please refer to Table S2.

## 4 DISCUSSION

Our study clearly shows that focal epilepsy and temperature‐related seizures may be part of the AGAT‐related spectrum. Specifically, we found two of four siblings carrying the homozygous pathogenic AGAT:c.446>A:p.Trp149* variant who developed focal epilepsy with late adolescence or adulthood onset. Additionally, three of four affected family members experienced febrile seizures plus and temperature sensitivity. Notably, no other plausible causes of epilepsy were identified in the affected individuals. Moreover, taking a broader perspective, epilepsy has been reported in more than half of individuals with alternative creatine deficiency disorders caused by pathogenic variants in other genes, such as SLC6A8 and GAMTPEVuZE5vdGU.^4^, ^14^ Therefore, it is reasonable to infer that epilepsy may also be a component of the AGAT‐related spectrum.

The AGAT gene has not yet been recognized as an epilepsy‐related gene in the most up‐to‐date and curated databases, such as Gene4Epilepsy^8^ or the GEL panel (v7.46).^9^ Furthermore, epilepsy has not been reported as part of its phenotype in the Online Mendelian Inheritance in Man catalogue.^15^ Of course, additional independent reports involving families beyond this one would be of interest and could support the inclusion of this gene in dedicated genetic panels.

We also observed fever sensitivity in seizure occurrence in our AGAT‐deficiency family. Specifically, three of four affected individuals reported febrile seizures up to the age of 12 years and experienced worsening seizures with temperature. We consider these findings particularly relevant in the context of climate change. We have recently highlighted the complex relationship between neurological disorders, including epilepsy, and climate change.^16^ These anecdotal observations may contribute to the scientific community's efforts to develop preventive strategies, even for rare neurodevelopmental disorders.

We are particularly intrigued by the strictly “sensory” seizure semiology exhibited by the proband and her brother, characterized by limb or tongue tingling or ictal parietal headache. This type of seizure semiology suggests a primary involvement of the parietal somatosensory cortex. We acknowledge that the proband's brother experienced seizures only during early adolescence, and we recognize that detailed semiology within families may sometimes be influenced by other family members. Unfortunately, we were unable to record an ictal electrographic seizure. However, interictal EEG findings in the proband are consistent with parietal involvement. It is important to note that simple aware sensory seizures can be easily overlooked, potentially leading to a delayed or missed diagnosis of epilepsy. Moreover, Abdennadher et al.,^14^ in a series of 18 cases with SLC6A8 deficiency, reported sensory seizures in one case and temporoparietal discharges in three individuals. We believe that the cortical somatosensory involvement as a key region in creatine disorders has a solid biological plausibility in our cases, as demonstrated by *Z*‐score atrophy and AGAT mRNA expression maps, indicating selective atrophy and lower gene expression in postcentral regions, respectively.

The implications of a missed diagnosis of epilepsy related to AGAT deficiency disorder are substantial, as a tailored therapy involving oral creatine supplementation is available. This treatment has been shown to prevent adverse neurodevelopmental outcomes, as previously reported.^3^ As an example, in our family, the proband's brother, who began creatine replacement therapy when he was 4 months old, has no intellectual disability, in contrast to the proband, who started treatment at the age of 4 years.

We also identified corpus callosum mild dysmorphic features in three individuals. We propose that corpus callosum abnormalities may be part of the AGAT‐related disorder and could potentially contribute to epileptic seizures. Corpus callosum abnormalities have also been reported in other creatine deficiency disorders, such as those associated with the GAMT gene.^17^ Moreover, in a comprehensive study involving 101 individuals with SLC6A8 deficiency, 53 of the 73 for whom brain MRI data were available exhibited abnormalities, such as a thin corpus callosum.^18^ Additionally, experimental studies on mice with AGAT‐ and GAMT‐related creatine deficiency have suggested that corpus callosum abnormalities are key neuroanatomical features of creatine deficiency disorders.^19^ Specifically, mice with homozygous AGAT deletion exhibited a significant reduction in corpus callosum volume compared to wild‐type mice.^19^ It is also known that creatine deficiency disorders may specifically impair the timing of oligodendrocyte myelination^20^; such findings further support the biological plausibility of the corpus callosum dysmorphisms observed in our cases.

We were able to provide potential answers to the first two research questions of this study. However, we do not claim to fully address the final question: is there evidence of neurodegeneration in adult individuals with AGAT deficiency despite creatine supplementation therapy? Our study has several limitations, with only a cross‐sectional design and a limited number of cases. Nonetheless, we believe we can offer some insights into this issue.

First, two of four affected individuals developed epilepsy with the same electroclinical phenotype after many years of oral creatine supplementation. Second, it is well established that oral creatine supplementation does not fully restore brain creatine levels, as demonstrated by MRS studies. Third, our advanced MRI analysis revealed severe widespread cortical thinning, when compared to a control group. Thus, we believe it is reasonable to ask: is oral creatine supplementation sufficient, or should alternative delivery strategies be considered? Of course, further studies are warranted.

## AUTHOR CONTRIBUTIONS

**Francesco Fortunato**: Writing—original draft preparation; conceptualization; data curation; formal analysis; investigation; methodology; supervision. **Roberta De Fiores**: Data curation; formal analysis; investigation; methodology; writing—original draft preparation. **Ilaria Sammarra**: Data curation; formal analysis; investigation. **Miriam Sturniolo:** Data curation; methodology. **Iolanda Martino:** Data curation; investigation. **Maria Celeste Bonacci:** Formal analysis; data curation; investigation. **Maria Eugenia Caligiuri:** Formal analysis; data curation; investigation. **Antonio Gambardella:** Conceptualization; data curation; investigation; methodology; supervision; writing—review & editing; funding acquisition.

## FUNDING INFORMATION

This work was supported by #NEXTGENERATIONEU and funded by the Ministry of University and Research, National Recovery and Resilience Plan, project MNESYS (PE0000006)—A Multiscale Integrated Approach to the Study of the Nervous System in Health and Disease (DN. 1553 11.10.2022).

## CONFLICT OF INTEREST STATEMENT

None of the authors has any conflict of interest to disclose. We confirm that we have read the Journal's position on issues involved in ethical publication and affirm that this report is consistent with those guidelines.

## ETHICS STATEMENT

This survey has been approved by our institution's ethics committee and has been performed in accordance with the ethical standards laid down in the 1964 Declaration of Helsinki and its later amendments. We obtained patients' informed consent, and data were treated according to the European General Data Protection Regulation 2016/679.

## Supporting information


Data S1.

## Data Availability

Deidentified data that support the findings of this study are available upon request to the corresponding author.
