# Supplementary material for: Focal epilepsy with sensory seizures associated with arginine:glycine amidinotransferase deficiency: A clinical and advanced magnetic resonance imaging study
Source: Epilepsia. 2025 May 5;66(7):e136–41. doi: 10.1111/epi.18442 (PMC12291002; doi:10.1111/epi.18442)
Supplement: Supplementary file 1 — Data S1. [file EPI-66-e136-s001.docx]

**Supplementary Information:**

**1) Supplementary Materials**

**2) Supplementary Figure**

**3) Supplementary Tables**

**4) References**

**Supplementary Material 1: Electrophysiological and brain magnetic resonance imaging assessment**

Each individual underwent a dedicated epilepsy assessment which comprehends both EEG and 3T-brain-magnetic resonance imaging (MRI) assessment. Standard EEG recording with hyperventilation and photic stimulation were performed according to the latest International Against Epilepsy League (ILAE) recommendations^1^. EEG recordings were classified as either normal or showing either interictal epileptiform discharges (IEDs) or abnormal slowing by two blinded neurologists with specialized expertise in epilepsy (I.S. and F.F.).

All subjects underwent brain MRI using the same 3T MR750 General Electric scanner with an eight-channel head coil (Discovery MR-750, GE, Milwaukee, WI, USA).

Brain MRI with 3T scan was performed according to the latest ILAE Harmonized Neuroimaging of Epilepsy Structural Sequences (HARNESS-­ MRI) protocol^2^. This protocol includes a 3D-T1-weighted spoiled gradient echo sequence (sagittal acquisition, TE/TR = 3.7/9.2 ms, matrix size 256 × 256, flip angle = 12°, isotropic voxel = 1 × 1 × 1mm3). FreeSurfer (v 7.2) (http://surfer.nmr.mgh.harvard.edu) was used to perform automated brain morphometric analysis on 3D-T1-weighted images and obtain, using the standard pipeline^3, 4^ , the following measures: cortical thickness from 34 gray matter regions for each hemisphere. Outputs were quality checked by visual inspection followed by quantitative identification of outlier volumes which significantly deviate from the group mean. The analysis was conducted by two brain scientists (M.E.C. and M.C.B.) with specialized expertise in neuroimaging of epilepsy. For each individual case with AGAT deficiency, we calculated Z-scores for each region of interest (ROI) in the quantitative MRI analysis using a dataset consisting of 30 age- and sex- matched healthily individuals [18 females; 28.2± 3.7 years]. We then computed the average value between the left and right sides for each ROI. Maps of brain Z-scores were calculated using the residuals from the control cohort with a general linear model, formulated as follows: ‘ROI ~ Age + Gender + Intracranial Volume (ICV)’.

**Supplementary Material 2: Allen Human Brain Atlas, brain expression map of the AGAT Gene – pipeline.**

We used Allen Human Brain Atlas (AHBA) (<https://human.brain-map.org>)^5^ as the primary source of gene-expression data. The AHBA sampled six donor brains (1 female, age 24-57 years) and quantified mRNA expressions, also providing the Montreal Neurological Institute (MNI) coordinates for each sample^5^. To reduce the complexity of the AHBA data, we summarized the data into the Desikan–Killiany cortical atlas which was built into the FreeSurfer software for automatic labeling of regions of interest, employing a validated pipeline previously published^6^.

The obtained map was then compared with the cortical thickness maps obtained from the recruited cases, enabling a comprehensive evaluation of potential correlations between gene expression patterns and structural brain variations.

**Supplementary Figure 1: Family Pedigree**

**
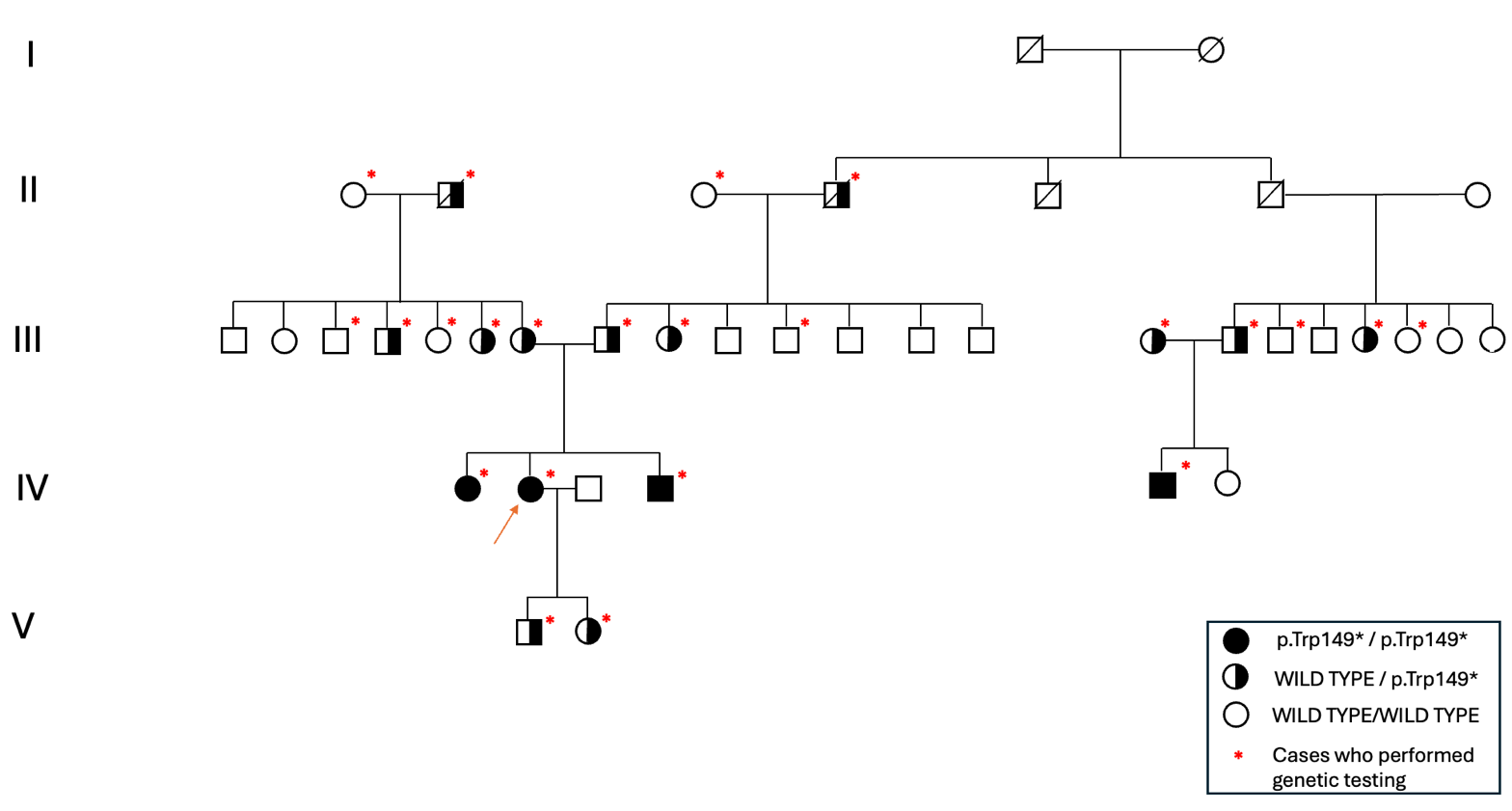
**

**Footnotes:** p.Trp149* is the variant in the AGAT gene

**Supplementary Table 1: Demographic and clinical features of cases with AGAT deficiency**

| **Case, Gender** | **Age at observation (years)** | **AGAT variant**  **(NM_001482.3)** | **Febrile seizures onset (months)** | **First epileptic seizure (years)** | **Prenatal and perinatal history** | **Developmental milestones** | **Intellectual disability** |
| --- | --- | --- | --- | --- | --- | --- | --- |
| IV1, F | 33 | c.446>A: p.Trp149* | - | - | Unremarkable | began to walk at 24 months.  first words at 30 months | Moderate |
| IV2, F | 30 | c.446>A: p.Trp149* | 12 | 30 | Unremarkable | began to walk at 24 months.  first words at 30 months | Moderate |
| IV3, M | 20 | c.446>A: p.Trp149* | 72 | 12 | Unremarkable | Unremarkable | - |
| IV4, M | 24 | c.446>A: p.Trp149* | 18 | - | Unremarkable | began to speak simple sentences at around 36 months | Mild |

*Footnotes: AGAT: arginine:glycineamidinotransferase;F= female, M= male;*

**Supplementary Table 2: Allen Human Brain Atlas, brain expression map of the AGAT Gene – results**

| **Cortical Region of Interest** | **AGAT mRNA expression** |
| --- | --- |
| **ctx-lh-caudalanteriorcingulate** | 9.23061304285884 |
| **ctx-lh-bankssts** | 9.22423915332122 |
| **ctx-lh-temporalpole** | 9.10502832659831 |
| **ctx-lh-isthmuscingulate** | 9.03912815819352 |
| **ctx-lh-parahippocampal** | 9.01699596544955 |
| **ctx-lh-rostralanteriorcingulate** | 8.97117602965415 |
| **ctx-lh-caudalmiddlefrontal** | 8.96491809968082 |
| **ctx-lh-parsorbitalis** | 8.96194791678044 |
| **ctx-lh-superiorfrontal** | 8.94051011537062 |
| **ctx-lh-frontalpole** | 8.93851910095559 |
| **ctx-lh-medialorbitofrontal** | 8.91192097436332 |
| **ctx-lh-inferiortemporal** | 8.8956614838008 |
| **ctx-lh-middletemporal** | 8.88877098612806 |
| **ctx-lh-parstriangularis** | 8.86157838115791 |
| **ctx-lh-lateralorbitofrontal** | 8.85452890345813 |
| **ctx-lh-rostralmiddlefrontal** | 8.85178665089039 |
| **ctx-lh-precentral** | 8.84777858189694 |
| **ctx-lh-fusiform** | 8.83978187110335 |
| **ctx-lh-parsopercularis** | 8.83090038640532 |
| **ctx-lh-entorhinal** | 8.80043644844275 |
| **ctx-lh-posteriorcingulate** | 8.79850021713936 |
| **ctx-lh-superiortemporal** | 8.7914233782777 |
| **ctx-lh-supramarginal** | 8.78696234601406 |
| **ctx-lh-precuneus** | 8.78187152658185 |
| **ctx-lh-postcentral** | 8.77540341261487 |
| **ctx-lh-insula** | 8.73644779181727 |
| **ctx-lh-superiorparietal** | 8.71849931776373 |
| **ctx-lh-inferiorparietal** | 8.70431775318766 |
| **ctx-lh-transversetemporal** | 8.68457256039004 |
| **ctx-lh-paracentral** | 8.67817943889796 |
| **ctx-lh-cuneus** | 8.52724248906295 |
| **ctx-lh-lateraloccipital** | 8.46393202935277 |
| **ctx-lh-lingual** | 8.4468222833698 |
| **ctx-lh-pericalcarine** | 8.25017199394338 |

**Footnotes:** Ranked expression levels of AGAT mRNA generated via Allen Human Brain Atlas, from regions of interest (ROIs) with higher expression to those with lower expression.

**4) References**

1. Peltola ME, Leitinger M, Halford JJ, Vinayan KP, Kobayashi K, Pressler RM, et al. Routine and sleep EEG: Minimum recording standards of the International Federation of Clinical Neurophysiology and the International League Against Epilepsy Epilepsia. 2023 Mar;64:602-618.

2. Bernasconi A, Cendes F, Theodore WH, Gill RS, Koepp MJ, Hogan RE, et al. Recommendations for the use of structural magnetic resonance imaging in the care of patients with epilepsy: A consensus report from the International League Against Epilepsy Neuroimaging Task Force Epilepsia. 2019 Jun;60:1054-1068.

3. Fischl B. FreeSurfer Neuroimage. 2012 Aug 15;62:774-781.

4. Labate A, Caligiuri ME, Fortunato F, Ferlazzo E, Aguglia U, Gambardella A. Late drug-resistance in mild MTLE: Can it be influenced by preexisting white matter alterations? Epilepsia. 2020 May;61:924-934.

5. Hawrylycz MJ, Lein ES, Guillozet-Bongaarts AL, Shen EH, Ng L, Miller JA, et al. An anatomically comprehensive atlas of the adult human brain transcriptome Nature. 2012 2012/09/01;489:391-399.

6. French L, Paus T. A FreeSurfer view of the cortical transcriptome generated from the Allen Human Brain Atlas Front Neurosci. 2015;9:323.
